# Advancements in Machine Learning and Artificial Intelligence in the Radiological Detection of Pulmonary Embolism

**DOI:** 10.7759/cureus.78217

**Published:** 2025-01-29

**Authors:** Maneeshaa Mohanarajan, Prachi P Salunke, Ali Arif, Paola Melissa Iglesias Gonzalez, David Ospina, Dario S Benavides, Chaithanya Amudha, Kumareson K Raman, Humza F Siddiqui

**Affiliations:** 1 Integrated Urgent Care, London Ambulance Service, London, GBR; 2 Medicine and Surgery, Tver State Medical University, Tver, RUS; 3 Medicine, Dow University of Health Sciences, Karachi, PAK; 4 General Surgery, Doctor Arnulfo Arias Madrid Hospital Complex, Panama City, PAN; 5 Internal Medicine, Universidad de los Andes, Bogotá, COL; 6 General Medicine, Universidad del Rosario, Bogotá, COL; 7 Medicine and Surgery, Saveetha Medical College and Hospital, Chennai, IND; 8 Cardiology, Nottingham University Hospitals National Health Service (NHS) Trust, Nottingham, GBR; 9 Internal Medicine, Jinnah Postgraduate Medical Centre, Karachi, PAK

**Keywords:** artificial intelligence, computed tomography pulmonary angiography, convolutional neural networks (cnn), machine learning, pulmonary embolism, radiological imaging

## Abstract

Pulmonary embolism (PE) is a clinically challenging diagnosis that varies from silent to life-threatening symptoms. Timely diagnosis of the condition is subject to clinical assessment, D-dimer testing and radiological imaging. Computed tomography pulmonary angiogram (CTPA) is considered the gold standard imaging modality, although some cases can be missed due to reader dependency, resulting in adverse patient outcomes. Hence, it is crucial to implement faster and precise diagnostic strategies to help clinicians diagnose and treat PE patients promptly and mitigate morbidity and mortality. Machine learning (ML) and artificial intelligence (AI) are the newly emerging tools in the medical field, including in radiological imaging, potentially improving diagnostic efficacy. Our review of the studies showed that computer-aided design (CAD) and AI tools displayed similar to superior sensitivity and specificity in identifying PE on CTPA as compared to radiologists. Several tools demonstrated the potential in identifying minor PE on radiological scans showing promising ability to aid clinicians in reducing missed cases substantially. However, it is imperative to design sophisticated tools and conduct large clinical trials to integrate AI use in everyday clinical setting and establish guidelines for its ethical applicability. ML and AI can also potentially help physicians in formulating individualized management strategies to enhance patient outcomes.

## Introduction and background

Pulmonary embolism (PE) is one of the most clinically challenging medical emergencies due to its often silent but potentially life-threatening nature, prompting physicians to seek faster and more precise diagnostic tools in order to reduce the associated morbidity and mortality. The mortality of PE can be as high as 30% in undiagnosed cases, and around 8% in patients who are promptly diagnosed and treated [1]. PE has an incidence of approximately 60 to 70 cases per 100,000, which has been steadily increasing in recent years due to a surge in risk factors. A study reported by Shi-Hsien Hsu et al. evaluated the incidence of PE in emergency rooms (ER) finding that the visits due to PE increased by 319,000 from 2010 to 2012 and 441,000 from 2017 to 2018 [2]. Studies such as computerized tomography (CT) and CT pulmonary angiography (CTPA) are considered the most helpful diagnostic imaging tests for PE, but their complex interpretation usually requires trained radiologists who may not always be readily available. The high burden on radiologists and the lack of other trained personnel to perform and interpret these interventions pose additional challenges for the early assessment and diagnosis of PE. The current diagnostic protocols and tests for PE, although proven useful, continue to face challenges and limitations, especially in diagnosing segmental and subsegmental PEs, and have a scope for error due to inter-rater dependency. Hence, the necessity for alternative innovative tools has surged with the implementation of novel technologies [3, 4].

**Figure 1:**
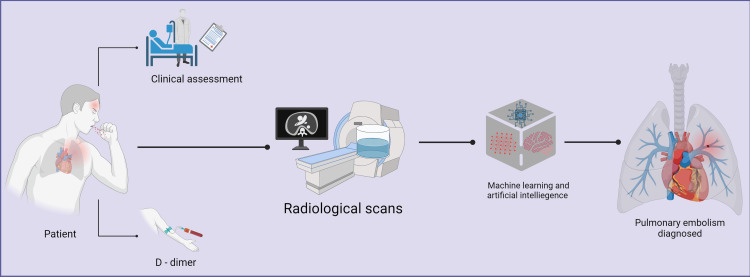
Summary of use of artificial intelligence and machine learning in radiological detection of pulmonary embolism Image credit: Humza F. Siddiqui. Created using biorender.com.

The emergence of artificial intelligence (AI) in the health sector is set to revolutionize the way in which physicians interpret and diagnose diverse clinical conditions, marking a new era for medicine. In recent years, research studies and scientific publications evaluating the use of AI have increased dramatically in virtually all fields of medicine, with diagnostic radiology being no exception. Analytic AI tools are promising, high-yield clinical appliances for optimizing not only the diagnosis but also the risk stratification and personalized management of diseases that are commonly evaluated through radiologic practices, including PE. The implementation of AI in medical imaging has potentially resulted in reduced reading time, more efficient workflow, decreased need for contrast agents or lower dosing requirements as well as a minimization of human error. AI has a wide potential for clinical research and may soon become an essential aid for physicians, with the main goal of improving outcomes in clinical entities such as PE [5, 6] (Figure 1).

Several AI tools have been designed, proposed and continue to be investigated for the assessment of PE [7, 8]. These instruments operate mainly on computer-aided design, a broad term that encompasses machine learning (ML) or deep learning (DL). ML can be used to formulate an image processing technology that can contribute as a non-human “second opinion” for the physician [9]. Computer-aided design software can help improve diagnosis, prognosis and management of PE as well as facilitate data collection and analysis, generation of educational materials and feedback for healthcare professionals. Although many of these operational systems have not been peer-reviewed, regulating agencies such as the Food and Drug Administration (FDA) and European Commission (EC) have approved their marketing and are thus presently utilized by many healthcare institutions. The limited legislation for their use has already been enacted [10]. This narrative review explores the evidence on currently available AI and ML tools and their impact and efficacy in the radiological diagnosis and evaluation of PE.

## Review

Diagnosis and radiological modalities of pulmonary embolism

PE is a serious, potentially fatal condition caused by the obstruction of the pulmonary artery or its branches, most commonly as a consequence of deep vein thrombosis (DVT). The pathophysiology of PE is explained by Virchow's triad, which outlines three primary factors: blood stasis, vascular injury, and hypercoagulability [11, 12]. Around 30% of PEs are incidentally detected during pulmonary workups, while patients can present with nonspecific or life-threatening symptoms. Acute pleuritic chest pain is the most common symptom of PE. Dyspnea and hemoptysis are frequently associated with PE, occasionally accompanied by cough, stridor or wheezing, up to one-third of patients with acute PE exhibit nonspecific symptoms, including syncope, dizziness, tachypnea, tachycardia, hypo- or hypertension, diaphoresis, and leg swelling. In patients with low or moderate clinical probability, a negative D-dimer test can help rule out PE [13].

Diagnosis of PE requires a multidisciplinary approach, starting with a clinical examination and patient history. Non-invasive tests including chest X-rays, echocardiography and electrocardiograms, and blood tests including D-dimer and arterial blood gases, supplemented with more advanced imaging. Ventilation-perfusion scintigraphy and computed tomography angiography confirm the diagnosis. Despite advances in diagnostic tools and interventions, PE remains underdiagnosed and a significant preventable cause of in-hospital mortality [14]. Estimating the pre-test probability of PE is critical to identifying patients at high clinical risk [15]. The Wells and Geneva scores, based on the patient's clinical characteristics, are commonly used to assess this probability. PE ranks as the third most common acute vascular disease after myocardial infarction and stroke [16]. Its presentation varies widely, making it difficult to diagnose, although potentially fatal. CT pulmonary angiography (CTPA) and lung scintigraphy are the primary imaging methods for diagnosing PE. Each method has its advantages and limitations, with specific cases favoring one over the other. CTPA identifies the clot within the pulmonary vasculature, whereas ventilation-perfusion scintigraphy evaluates the functional ramifications of the clot. Earlier both ventilation-perfusion (V/Q) scan and CTPA were used in a similar ratio for suspected PE diagnosis. However, in the last decade, the use of CTPA has substantially increased in comparison to V/Q scans [17, 18]. Scintigraphy is advocated under circumstances in which radiation exposure needs to be avoided such as pregnancy [19].

CT Pulmonary Angiography

CTPA is increasingly favored as a first-line diagnostic tool for suspected PE due to its wide availability, high diagnostic accuracy, and lower morbidity compared to traditional pulmonary angiography. Technical advancements have enhanced its sensitivity and specificity, making it highly effective. The procedure involves administering intravenous non-ionic contrast via a peripheral vein, with 1-1.5 mm slices captured from the thoracic inlet to the diaphragm during a single breath-hold. It can also visualize cardiac structures and assess right-to-left ventricle ratios and pulmonary artery dimensions [20].

The limitations of CTPA include radiation exposure, potential nephrotoxicity from iodinated contrast, and high cost [21]. The key challenges include distinguishing between true emboli and artifacts and differentiating non-PE findings like the "pulmonary wedge appearance" or an endoluminal ring in the azygous vein [22]. CTPA remains a reliable diagnostic tool, with excellent specificity, sensitivity, and negative predictive value for both massive and non-massive PE. Although CTPA is the gold standard for diagnosing PE, its use is limited by cost, contrast-related risks, and availability, especially in emergency settings. In pregnant patients, alternative imaging is recommended due to radiation concerns [23].

V/Q Single-Photon Emission CT (SPECT)

Planar V/Q scintigraphy has been a standard diagnostic tool for assessing PE, particularly in evaluating the lower lung fields. However, its effectiveness can be limited by the low resolution of the gamma camera, making interpretation of ventilation and perfusion defects challenging. The introduction of single-photon emission computed tomography (SPECT) offers enhanced resolution and clearer assessment of areas of interest. Studies have shown that SPECT achieves high agreement with conventional V/Q imaging in detecting mismatched defects in the lungs, accurately characterizing in 16 out of 18 patients. Despite its advantages, SPECT can tend to undergrade ventilation defects and over grade perfusion abnormalities, similar to the limitations of planar V/Q scintigraphy. Nonetheless, it proves useful in complex cases, particularly those involving middle or lower lung zones [24]. High-probability V/Q SPECT has emerged as a valuable alternative for diagnosing PE, capable of accurately assessing most cases with a single scan. When combined with conventional V/Q scanning and clinical prediction rules, it enhances the evaluation of intermediate-risk patients. Concerns regarding radiation exposure, longer operational time, need of superior technical interpretation skills and expensive infrastructure pose challenges to the wide use of the modality [25].

Magnetic Resonance Angiography

Magnetic resonance angiography (MRA) effectively detects emboli in medium-sized vessels and provides valuable insights into distal circulation. It is especially useful for patients who cannot tolerate contrast agents or have impaired renal function, as it does not require contrast. MRA is also advantageous for individuals with larger body surface areas, offering more contrast sequences compared to X-ray computed tomography (CT). Ultra-high field systems allow for detailed visualization of emboli in the distal lung arteries, and the technology is not limited by test duration or patient condition, making it a potential alternative diagnostic tool for PE diagnosis [26].

Magnetic resonance imaging (MRI) and other advanced imaging techniques facilitate the diagnosis of PE, particularly in complex cases such as emboli present in unusual locations like smaller peripheral arteries, potentially managing workload. The advent of deep learning algorithms has further enhanced the prediction of high-risk PE and associated mortality, offering potential improvements in screening, diagnosis, monitoring, and prognosis. However, MRI for pulmonary angiography is not always feasible due to limitations such as claustrophobia, pregnancy, and the presence of implanted devices like pacemakers [27].

Role of artificial intelligence (AI) in medical imaging

Medical imaging encompasses various modalities, each of which presents a static or dynamic representation of the human anatomy and its operation in health and disease [28-30]. AI is the broader concept, which involves the emulation of human tasks such as reasoning, visualization, understanding language, and learning. Its application in the field of medical imaging refers to the capability of the computer in identifying, studying, and processing medical imaging data for accurate, efficient, standardized, and reliable interpretation [31]. The integration of AI in medical imaging has the potential to revolutionize the dimension of healthcare with better patient outcomes by improving disease detection, disease characterization, predicting prognosis, and customizing treatment through AI-driven precision medicine [32-35].

There have been many studies on radiological expertise and its relationship with visual perception, presenting that diagnostic errors can occur due to human limitations in both conscious and unconscious perceptions, cognition, and judgment. False-positive and -negative results, variations caused by readers, disease prevalence, and patient factors all compromise the benefit of medical imaging. Known techniques for reducing these variations include double or consensus reporting, as well as positioning and protocol guidelines on the acquisition side. To curtail human error in diagnostic imaging, it is necessary to provide high-quality images in a timely manner, and human error in diagnosis is mitigated through the use of computer-aided diagnostic systems. Other challenges are having less time to interpret the images and the increasing size of the patient cohort to evaluate new images. The latter stems from the aging population and the increasing access to the latest imaging modalities. Given these hurdles, new ideas and approaches in medical imaging are necessary to address these challenges and continue to advance and investigate new approaches and technologies [36-39].

Interpretation and Diagnosis

Image interpretation is the primary work of the radiologist, and the diagnostic decision-making requires summarizing multitudes of findings [40]. It is well known that both mental and physical fatigue of the radiologist or technologist in the practice of reading images reduces the accuracy of interpretation. Radiologists are also as variable as any other specialists in their expertise for particular items. Misinterpretation of imaging findings can lead to significant health care burdens [41]. Thus, it is not so surprising that the primary proposal of many medical applications of artificial intelligence has been to provide alerts or support in the decision-making process for diagnosis [42, 43].

Artificial intelligence tools have a broad range of applications in the field of medical imaging, including image reconstruction and enhancement, analysis, segmentation, and classification of images, and computer-aided diagnosis for providing diagnostic estimates. AI can enhance differentiation between malignant and benign lesions and objectively identify the dilated tissue. Early investigation may help to assess the outcome of surgical resection or treatment efficiency. Such tools continuously evolve as more annotated data become available and machine learning and deep learning improves. The capability of AI-based methods to learn from data will eventually result in tools able to provide information about survival rates and probable outcomes linked with quality of life, treatment, and patient outcomes [44]. AI was able to perform a task at least as well, if not better, than human observers, in this case distinguishing between low-grade and high-grade gliomas. In the context of nuclear medicine data, AI has been investigated to provide additional data derived from tumor heterogeneity, tumor perfusion, and tumor cellularity regarding the same exam. This is particularly important considering applications of this new tool linked to the arrival of specific treatments and new in vivo immune therapies [45].

Image Segmentation and Classification

Image segmentation is the task of partitioning an image into meaningful segments to allow easier extraction of essential information from it. Afterwards, the results may serve as an input for other image analysis applications such as shape modeling, patient-specific organ modeling, diagnosis, growth quantification, and therapy evaluation. Properly solved, it offers physicians additional, more precise data about health status, unveiling unusual or rare abnormalities visible in scans, and enabling more reliable diagnostics. With respect to MR angiography, perfusion, and diffusion modalities, proper image processing can also predict out-of-the-ordinary events or surprises [46].

Image classification refers to assigning a class label to an image based on the image content. Feature-based machine learning is a complicated yet helpful method that human experts are already proficient at when applied with a vast database of labeled exams. AI with machine learning and deep learning provides automatic and consistent performance at the same level of quality as humans do. Convolutional neural networks (CNN) classifiers give equal performance in distinguishing age, sex, and large-scale dementia or other brain diseases, and brain segmentation outputs. Within medical imaging, machine learning techniques are currently being used for many applications. For example, in invasive cardiology, machine learning methods proved their effectiveness in vessel tree segmentation, artery-vein differentiation, and ventricle segmentation [47]. In MR image processing, a combination of shallow and deep learning models was proposed to provide patients' image-text-based disease prediction. Among other applications, the attentional focused network for object recognition on the retinal fundus image and lung diseases, where AI classifiers enhanced the accuracy of disease diagnosis. For fetal MR imaging, the classification of brain volume is based on generative adversarial networks, and an automatic abnormal fetal brain identification is represented. Nevertheless, AI classifiers have difficulties in heterogeneous areas, while they require large, labeled datasets to learn from and decision models reflecting the true data characteristics in image scanning. Besides, biased AI classifiers often have a decrease in performance when there are more and more data from other scanners and other acquisition parameters [48].

Improved Accuracy and Efficiency

The integration of artificial intelligence (AI) techniques has been shown to enhance the diagnostic accuracy of various imaging modalities. This includes the tasks of detecting, classifying, and quantifying disease states. In addition to improved accuracy, AI in medical imaging offers the ability to process an enormous volume of data quickly, resulting in reduced turnaround times for clinical results. This, coupled with the ability to evaluate every imaging study in a standardized and objective manner, could lead to more reliable results with a high degree of confidence. This could lead to a number of metrics that would quantify the improvements in diagnostic accuracy possible with AI applications, such as higher sensitivity and specificity, lower false-negative and false-positive rates, and lower inter-operator variability. Improved accuracy and efficiency, in turn, could decrease morbidity, lead to earlier detection and treatment, reduce unnecessary biopsy rates, produce fewer missed lesions when reading non-contrast studies, reduce numerical interobserver variability in lesion staging, and minimize operator fatigue [39].

AI, or machine learning techniques, that can categorize images by recognizing textural, anatomic, or physiologic patterns might also reduce reader error at some levels. Interpretive errors are difficult to estimate but range from 1.1% to 3%, predominantly from missed cancers. Errors of commission in diagnostic imaging are estimated at 2% and are higher in primary interpretation compared with subsequent interpretations [49]. Stemming from biomedicine, artificial intelligence has evolved from traditional boundary demarcation for diagnosing disease states and is moving into fields never before touched by traditional data. With the evolution of imaging into multidimensional data sets, automation of tasks such as interpreting the shadows in an imaging study goes a step further. AI has the potential to reduce some of the inter-operator errors that occur when summarizing lesions, as there are data that describe high levels of variability among radiologists in summarizing lesion CT attenuation coefficients, thus influencing tumor node Metastasis staging and affecting treatment planning and predicting outcomes. Specifically, deep learning is the newest method for AI [50]. Deep learning applications have the potential to revolutionize imaging in health care and could automatically detect and identify, triage, and quantify without human engineering from a variety of imaging studies. The most current work has only grazed the waves of what might be available and possible by using deep learning in imaging [51].

Implementation of computer-aided design and machine learning in pulmonary embolism

Several novel models of machine learning (ML) have been implemented in the detection of pulmonary embolism using radiological images. Models trained and developed using existing datasets are often looked at retrospectively to investigate the validity and usefulness of ML.

Gotta et al. [52] investigated the use of ML in diagnosis and prognosis of PE, by identifying first the radiomic features from dual-energy computed tomography (DECT) scans, then inputting the data into training of a “gradient boosted tree model” and testing the model. The authors conducted two studies on using this model. First, a sample size of 107 patients, where the ML model achieved a PE detection accuracy of 0.9 and area under curve (AUC) of 0.59, and second with a sample size of 131 patients' data with a high detection accuracy of 94% and with an AUC value of 0.91. Further statistical analysis was conducted using a Cox regression analysis to identify the significance of radiomic features to prognosis showing a c index of 0.991, correlating to high agreement of predicted and observed results. However, the extrapolation of this data to the general population will be difficult as the data is from a single center and does not account for clinical factors which in practice are often used to indicate the need for further radiological investigation [52, 53]. Feretzakis et al. tested a different model, Ultralytics YOLOv8, to check PE detection in COVID-19 patients with a sample size of 746 CTPA images. The model had a maximum mean average precision (MAP) of 0.85949, which varied at different stages in the testing. Although the model shows promise in the detection of PE, it will need further training to reach a consistently high accuracy rate [54].

Condrea et al. trained their model in segments using a dual-hop deep neural net as a framework. They used the CTPA scans of 12,012 patients (from RSPECT database) to help the model learn the anatomical structures as regions of interest. The accuracy of PE detection was indicated by a sensitivity of 92% and specificity of 96.1%. The introduction of an extra stage of training to make the model more anatomically aware seems to have increased the performance of the model overall [55]. Another model tested on two different PE datasets, Ferdowsi University of Mashhad’s pulmonary embolism (FUMPE) and computer-aided design PE (CAD-PE) by Hemalakshmi et al. shows even greater accuracy in the detection of PE of 99.75% in FUMPE and 99.83% in CAD-PE, respectively. The study uses a 2D UNet to generate a segmentation map, then applies further modification to the data to help detect if a CTPA image slice has PE or not. This model presents as a better model compared to the current best due to its multitasking ability using multihead attention (MHA) mechanism and specific inputs to provide more focused segmentation of CTPA image with consequent accurate detection of whether PE is present in said image or not [56].

The Lanza et al. study looks at the performance of nnU-Net algorithm in detecting PE, with correlation to blood clot volume (BCV) and right ventricle (RV) overload. The algorithm was tested on the RSPECT dataset for validation of diagnostic accuracy. The AUC values (and PE diagnostic accuracy) were 0.865 (83%), 0.937 (91%), and 0.848 (79%), respectively, although the algorithm seems to have limitations when dealing with more complex PE cases or detecting small peripheral emboli [57]. The study conducted by Aydin et al. also used a PyTorch U-Net to train and test an ML model using 1,550 patients’ data, with 2,339 CTPA images analyzed. The results showed an AUC of 0.88 and F1 score of 0.94, with high sensitivity (0.95) and precision (0.93). Therefore, indicating the segmentation of imaging, a U-Net algorithm could be a key part in the accurate diagnosis of PE in CTPA scans using ML models. A limitation of this study is the non-inclusion of clinical or lab results in the training of the ML model [58]. Additionally, Pu et al. investigated the use of a ML model with 3D recurrent residual U-net (R2 U-Net) to segment PE from CTPA scans, training it on both scans with and without manual annotation using a five-fold cross-validation method. The results showed PE detection with a Dice coefficient of 0.676±0.168 (with annotations) vs 0.647±0.192 (without annotations), with false-positive rate of 1.86 vs 4.20 per CT scan, respectively [59].

A study by Langius et al. shows a deep learning (DL) algorithm, that when tested with external data, actually had lower diagnostic accuracy than when tested with RSPECT. Langius-Wiffen et al. retrained and validated a deep learning (DL) algorithm retrospectively, with data (single-detector row CT (SDCT) scans converted to conventional images or virtual monochromatic images and multidetector-row CT (MDCT)) from two hospitals. The lower accuracy of diagnosis was found on conventional and VMI scans (originally SDCT) compared to MDCT scans, 81.8% and 72.7% compared to 93.9%, respectively [60]. Concurrently, Djahnine et al. study looks at the use of a 3D convolutional neural network (CNN) to analyze CTPA in the PE detection of 1268 patients from the CAD-PE and FUMPE databases. They used the following classifications: PE blood clot segmentation, PE classification, the Qanadli score, and right ventricle/left ventricle (RV/LV) prediction. The model worked well with an AUC of 0.87 in PE detection. In addition, the inclusion of Qanadli score and RV/LV diameter ratio was shown to be useful in the potential prediction of the severity of PE, as there was a strong correlation seen between the Qanadli score and total volume of blood clots in the regression analysis, with R2 score of 0.717 and 0.723. However, all the CTPA scans used in this study were “contrast enhanced”, hence this CNN may not perform as well with low-dose CT scans [61].

Huang et al. developed a 77-layer 3D CNN-denominated PENet and evaluated its effectiveness as a triage tool on PE from volumetric CTPA images. In this retrospective study, they collected 1797 CTPA studies from 1773 patients for the internal dataset and reported an area under the receiver operating characteristic curve (AUROC) of 0.84 [0.82-0.87], accuracy of 0.77 [0.76-0.78], sensitivity of 0.73 [0.72-0.74], and specificity of 0.82 [0.81-0.83]. The study used data from only one academic institution limiting universal generalization of the performance. Another limitation was the non-inclusion of cases of chronic or subsegmental PE since the focus was the model as an emergent triage tool [62]. Huhtanen et al. conducted a retrospective cohort study incorporating a deep neural network model denominated InceptionResNet v2 and a network with long-short term memory for processing. Additionally, there were two model versions using different pre-training data: Model A, created using over 100,000 chest X-ray provided by the National Institutes of Health dataset and Model B, utilized over 14 million natural images from the ImageNet dataset. Model A at stack and slice level achieved an AUROC of 0.94 and 0.97 and Model B at stack and slice level achieved an AUROC of 0.91 and 0.97 [63]. Khan et al. employed a deep learning framework based on DenseNet201 for the computer-aided diagnosis (CAD) of PE. This model categorizes the images using nine options: negative PE, indeterminate PE, right-sided PE, left-sided PE, central PE, RV/LV ratio>1, RV/LV ratio<1, acute PE and chronic PE CT. The results showed an accuracy of 88% [0.86-0.90], a sensitivity of 88% [0.86-0.90], a specificity of 89% [0.87-0.91] and an AUC of 0.90 [0.88-0.92]. An increase of 3% in AUC was observed with the latest model of CNN as compared to the AUC of the previous models, depicting marked improvement in accuracy of the algorithm. The authors suggested that possible future studies can use gradient-weighted class activation mapping (GRAD-CAM) to visualize the decision of the CNN model in each scan [64]. Table 1 summarizes all the studies.

**Table 1:** Summary of studies implementing computer-aided design and machine learning for the diagnosis of PE PE: Pulmonary embolism, CTPA: Computed tomography pulmonary angiography, DECT: Dual energy computed tomography, SDCT: Single-detector computed tomography, MDCT: Multi-detector computed tomography, VMI: Virtual monoemergic imaging, AUC: Area under the curve, ROC: Receiver operating characteristic, AUROC, Area under the receiver operating characteristic curve, CAD: Computer-aided design.

Author (Year)	Data set used	Machine Learning Model Used	Model Sensitivity	Detection Accuracy of PE	ROC and AUC values
Huang SC et al. (2020) [62]	CTPA	3D CNN	Stanford 0.73 Intermountain 0.75	Stanford 0.77 Intermountain 0.78	Data AUROC = 0.84 Ext. Data: AUROC = 0.85
Huhtanen H et al. (2022) [63]	X-rays and CTPA	InceptionResNet v2	Model a Stack level 86.6 Slice level 90.1 Model b Stack level 83.5 Slice level 90.8	Model a Stack level 90.2 Slice level 92.3 Model b Stack level 87.3 Slice level 90.1	Model A: Stack level 0.94 Slice level 0.97 Model B: Stack level 0.91 Slice level 0.97
Khan M et al (2023) [64]	CTPA	DL framework based on DenseNet201	0.88	0.88	0.90
Pu J et al (2023) [59]	CTPA	CNN -based segmentation and R2Unet	CNN = 0.62 R2 Unet = 0.57	CNN dice coefficient = 0.676 R2 Unet dice coefficient = 0.647	-
Aydın N et al (2023) [58]	CTPA	PyTorch U-Net	CTPA segmentation = 0.95	0.93	AUC = 0.88
Gotta J et al (2023) [52]	DECT	Gradient boosted tree model	Low risk PE = 0.50 Intermediate low-risk PE = 0.15 Intermediate high-risk PE = 0.98 High risk PE = 0.17	Low risk PE = 0.77 Intermediate low-risk PE = 0.77 Intermediate high-risk PE = 0.74 High risk PE = 0.90	Low risk PE AUC = 0.6 Intermediate low-risk PE AUC = 0.6 Intermediate high-risk PE AUC = 0.63 High risk PE AUC = 0.59
Hemalakshmi et al. (2024) [56]	CTPA	Modified PE - Ynet; using mainly nnUNet for multihead attention mechanism (MHA)	CAD-PE = 0.9885	FUMPE - 99.75% CAD-PE - 99.83%	AUC = 0.95
Langius -Wiffen et al. (2024) [60]	SDCT and MDCT	2020 RSNA challenge winning deep learning algorithm	MDCT Scan = 84.9% SDCT = 81.1% VMI = 82.7%	MDCT scans = 84.9% SDCT converted image = 81.8% VMI = 72.7%	MDCT AUC = 0.96 (95 % CI 0.92–0.98) SDCT converted image AUC = 0.89 (95 % CI 0.81–0.94) VMI AUC = 0.87 (95 % CI 0.80–0.93)
Lanza et al. (2024) [57]	CTPA	nnU Net algorithm	-	Overall = 83% Central PE was 91%	AUC = 0.865 (95 % CI 0.845–0.876) For Central PE AUC = 0.937 (95 % CI 0.920–0.954)
Feretzakis G et al. (2024) [54]	CTPA	Ultralytics YOLOv8 object detection model	-	Mean average precision (MaP) = 0.683	-
Gotta J et al (2024) [53]	DECT	Gradient-boosted tree model	1 (95% CI, 0.90-1.0, p = 0.0001)	0.94	AUC = 0.91
Condrea F et al. (2024) [55]	CTPA	Dual-hop architecture and Efficient Net V2-L	Sensitivity = 92.9% Specificity = 96.1%	87.3%	AUC = 0.6622
Djahnine A et al. (2024) [61]	CTPA	Retina U-Net	-	Dice coefficient = 0.784 R^2^ value = 0.723	AUC = 0.852

Implementation of artificial intelligence in the radiologic assessment of PE

The rate of missed incidental pulmonary embolism is variable but has been noted to be as high as 44.8%. However, the missed rate of pulmonary embolism substantially decreased to 2.6% with the use of AI tools. Studies have demonstrated that the missed diagnosis of pulmonary embolism, later detected by AI on retrospective analysis to be a soaring 38%. Artificial intelligence increases the diagnostic efficiency by increasing sensitivity and reducing time to interpret diagnostic imaging [65].

Ayobi et al. evaluated an AI application approved by the FDA called CINA-PE, which was used for the detection of pulmonary embolism (PE) on CT pulmonary angiography (CTPA). The AI demonstrated a high sensitivity and specificity of 93.3% and 94.8%, respectively. It had a positive predictive value of 89.5, reflecting an excellent detection rate and reliability. Missed rate was reduced from 15.6% to 3%, as the AI tool detected 29 more cases of PE initially missed by the radiologists. The retrospective selection of CTPA cases can lead to selection bias. The study did not compare the diagnostic performances of the software alone to that of single radiologists alone. It also did not evaluate the quality of images on the current dataset nor the percentage of optimal or suboptimal CTPAs for PE analysis [66].

Topff et al. compared the detection of incidental pulmonary embolism (iPE) with and without AI tool. The study revealed the diagnostic sensitivity of 91.6%, specificity of 99.8%, and accuracy of 99.6% of the AI tool. The iPE missed by radiologists considerably decreased from 44.8% to 2.6% with the implementation of AI software. However, the study had certain limitations. Historical controls as well as data sets were not all-inclusive of clinical cases, which generated data selection bias and underrepresented atypical presentations of iPE that derailed the diagnostic effectiveness of the AI. The quality of training for the AI hung precariously on the accuracy of the labelled data. Labelling and annotation bias was introduced as inconsistencies by radiologists in labeling could cause the AI to learn incorrect patterns of true-positives. Furthermore, the algorithm may also have algorithmic bias towards the more common imaging presentations that dominated the training data, failing to identify rare presentations and can potentially lead to feedback loop bias [67].

Ajmera et al. analyzed 251 CTPAs using a 2D segmentation AI model. The model accurately detected 44 out of 55 cases of PE with a sensitivity of 0.80, specificity of 0.74, and accuracy of 0.76. The validation of the model was performed on a single external test dataset instead of data sets from multiple clinical institutions to validate the model performance. Also, the model had a low positive predictive value on the external test dataset at both slice and scan levels. This means that when a test or diagnostic method yields a positive result, there is a low probability that the person actually has the condition or disease being tested for. The model is primarily built for detecting PE and all the scans should be interpreted by a radiologist to provide the final verdict. This human-in-the-loop approach is favored as it reduces the likelihood of missing or delay in reporting a positive scan [68].

Assessing a patient's risk of mortality is crucial when suspecting a diagnosis of PE. The right ventricular to left ventricular diameter ratio (RV:LV) is an important measurement in predicting adverse outcomes in patients with PE. A ratio of 1.0 or higher is a strong predictor of death. In a single-centered retrospective study with 101 consecutive patients with CTPA-proven acute PE, the maximal diameters were derived for RV:LV ratio using automated post-processing software and compared to manual analysis in two observers. Automated analysis led to a change in risk stratification in 45% of the patients (n=40). This information is vital to assess the severity in patients with suspected PE and will enhance patient outcomes [69].

The study performed by Hou et al. developed novel methods for predicting PE. The superior method the study established was the gradient boosting decision tree (GBDT) using machine learning. The outcome included an AUC of 0.799, sensitivity 63.9%, specificity 81.1%, and accuracy of 77.8%. The authors evaluated several algorithms, which include random forests, logistic regression, support vector machines, and scorecard analysis of relevant variables. Generally speaking, maximal D-dimer levels, plasma fibrinogen growth rates, and D-dimer growth rates were most critical features around the GBDT model. It proved that AI-based models, like GBDT, are efficient to advise doctors how to better monitor and handle the risk of PE for hospitalized patients [70].

Cheikh et al. conducted a cohort study on the diagnosis of pulmonary embolism demonstrated high performance for the AI algorithm as well as for emergency radiologists. A sensitivity of 0.926 and specificity of 0.958 was achieved by the AI algorithm, while a sensitivity of 0.9 and specificity of 0.991 was achieved by the radiologists. The AI found a total of 219 suspicious PEs that were all true-positives but with 14 false-negatives. The AI reported 180 suspicious number of cases of PE, out of which 171 were true-positives and nine false-negatives. More importantly, 19 truly positive PEs that the radiologists had not reported were identified by the AI. This analysis assessed the performance of the AI algorithm and radiologists under several subgroups established based on limiting factors to radiological interpretation including injection quality and respiratory artifacts. Such subgroups illustrated a consistent ability of the AI algorithm to maintain high sensitivity and specificity of reading by often matching or even outperforming that of radiologists [71].

AI tools have high diagnostic accuracy in detecting acute pulmonary embolism, but lower accuracy in chronic pulmonary embolism, requiring further research [72]. Deep learning models for PE detection attained pooled sensitivity and specificity rates of 0.88 and 0.86, respectively. These models analyzed imaging data mainly using convolutional neural networks (CNNs), which demonstrated great sensitivity but also highlighted issues with false-positives. The challenges remained in detecting chronic pulmonary embolism using AI. The literature on AI detection of CPE is mostly scant and scarce [73]. AI performance for CPE tends to be lower, standing at AUC of 0.69, whereas the values of acute PE range from 0.89 to 0.95 [74]. Vainio et al. utilized a novel technique of detecting chronic PE by analyzing the structure of pulmonary vessels 2D rotational maximum intensity projection (MIP) images generated from CTPAs by implementing CNN model and reported an accuracy of 0.89 [75]. Sadegh-Zadeh et al. performed a unique study and utilized diverse oversampling techniques to improve the performance of various machine learning models including artificial neural network (ANN), support vector machine (SVM), decision trees (DT), random forests (RF), and adaptive boosting (AdaBoost) for early mortality prediction. AI models can assist in post discharge care of PE. It aids in risk stratification, which involves evaluating each patient based on their unique data to determine which patients may be more susceptible to complications. RF proved to be superior over all other ML models in helping clinicians decide individualized optimal management strategies for each patient of acute pulmonary embolism [76]. Table 2 summarizes all the studies.

**Table 2:** Summary of AI tools used to detect PE radiologically AI: Artificial Intelligence, PE: Pulmonary embolism, CTPA: Computed tomography pulmonary angiography, CNN: Convolutional neural networks, AUROC: area under the receiver operating characteristic curve, ANN: artificial neural network, LR: logistic regression, SVM: support vector machine, DT: decision trees, GBDT: Gradient boosted decision trees, RV: right ventricle, LV: left ventricle.

Author (Year)	Dataset Used	AI tool Used	Outcome
Ayobi A et al. (2024) [66]	Anonymized chest CT pulmonary angiography (CTPA) scans together with their clinical reports.	CINA-PE CNN	The AI-based tool (CINA-PE v1.0.5) demonstrated a high performance, with a per-case sensitivity of 93.9% and a specificity of 94.8%. The algorithm was able to detect 22 out of the 29 cases (76%) that were initially missed by on-call radiologists, reducing the overall miss rate from 15.6% to 3.8%.
Topff L et al. (2023) [67]	The study used a dataset of 11,736 chest CT scans from 6,447 unique patients.	"AIDOC Medical" AI software.	The AI software was able to detect 96.3% of incidental pulmonary embolisms (IPEs) located in the main or lobar pulmonary arteries, including all main pulmonary clots. The use of the AI software resulted in a 94% reduction in missed IPEs by radiologists compared to the periods without AI assistance (44.8% vs 2.6%). The AI software had a median processing time of 3 minutes, with 99.5% of the results available to radiologists at the time they opened the study. The median detection and notification time (DNT) for IPE-positive examinations was reduced from 7714 minutes in the routine workflow without AI to 87 minutes with the AI-based worklist prioritization.
Ajmera P et al. (2022) [68]	251 CTPA scans	PE AI Screen with U-Net architecture.	The 2D segmentation U-Net model achieves high-performance metrics on the external test dataset, with a sensitivity of 0.80, specificity of 0.74, and AUROC curve of 0.85 at the scan level. The proposed model localized most of the pulmonary embolism cases correctly by achieving an average dice coefficient score for the test set as high as 0.743 ± 0.155 per scan, indicating high similarity between the reference and AI-predicted embolus masks. The standalone model took a mean time of 30.15 seconds to process the thin angiography sequences to make inferences and classify the scan as positive or negative for emboli, suggesting that the model is indeed fast with processing time. AI tool, "DxPE AI Screen", successfully promised rapid and reliable reporting among general radiologists and subspecialty-trained radiologists in the interpretation of CTPA scans for pulmonary embolism.
Seyed-Ali Sadegh-Zadeh et al. 2023 [76]	Computerized tomography (CT) angiography.	ANN, SVM, DT, AdaBoost and Random forest ML techniques	The study used various machine learning techniques, including ANN, SVM, DT, Random Forests, and Adaptive Boosting, to predict the mortality of patients with acute pulmonary embolism. The Random Forests model was found to be the best performing model in predicting mortality. The study highlighted the importance of using oversampling techniques to address the class imbalance issue in the dataset.
Hou L, et al. (2021) [70]	CT scans from 3619 patients.	XGBoost library for the GBDT model	The GBDT model demonstrated the best prediction with an AUC value of 0.799, whereas the RF model (AUC 0.791) was comparable, yet slightly weaker than the GBDT model. In contrast, the results of LR and SVM decreased significantly, yielding AUC values of 0.716 and 0.743, respectively. Sensitivity: The sensitivities of the risk prediction model (GBDT, LR, RF, and SVM) were 63.9%, 68.1%, 71.5%, and 75%, respectively. Specificity: The specificities of the risk prediction models were 81.1%, 66.1%, 72.7%, and 65.1%, respectively. Accuracy: The accuracies of the risk prediction models were 77.8%, 66.5%, 72.5%, and 67%, respectively.
Soffer S, et al. (2021) [73]	36,847 CTPA	CNN deep learning computer vision	The deep learning models achieved high sensitivities, ranging from 83% to 94.6%, with acceptable false positive rates and specificities ranging from 76.5% to 95.5%. One study reported an AUROC of 0.85 for a 3D CNN model, which was further improved to 0.95 by integrating imaging data with clinical data from the electronic health record.
Ma X, et al. (2022) [74]	Reports of ventilation-perfusion (V/Q) scans and CTPA studies	CNN	The focus of the study was on the development and evaluation of a convolutional neural network (CNN) algorithm for the analysis of pulmonary vasculature in 2D rotational maximum intensity projection (MIP) images to identify chronic pulmonary embolism (CPE).
Cheikh AB et al. (2022) [71]	CTPA from 1202 patients.	The AI algorithm used in this study was developed by AIDOC Medical, the algorithm was FDA- and EC-approved.	The accuracy for interpreting CTPAs in both the senior and junior radiologists was analogous, at 0.977 and 0.977 respectively; however, the performance of AI algorithm was inferior to the radiologists with an accuracy of 0.953 and 0.950, respectively, in their subgroups. The sub-cohort with poor injection quality was the one with the highest rate of discordance between the two radiologists and the AI algorithm, at 9% (6/67) of the cases. The very same sub-cohort has had also the highest discord between the gold standard and the radiologists, at 4.5% (3/67). For PEs missed by radiologists but not by the AI algorithm, the interpretation time was shorter, and they more commonly presented together with other confusing thoracic diseases and represented by short clot length in comparison with PEs missed by the AI algorithm but not the radiologists.

Discussion

Based on numerous studies on the topic of artificial intelligence (AI) and its functional role in detecting PE, a distinctive trend can be established. Many of the studies observed and tested the performance of a machine learning tool in conjunction with radiologists' findings. The use of AI demonstrated high performance in the detection of PE and showed a significant reduction in missed diagnoses and the overall miss rate by radiologists. Also, using AI-based software resulted in a substantial reduction in the median turnaround time for examinations showing evidence of PE. These AI-based tools have demonstrated superior sensitivity, specificity, accuracy, and negative predictive value compared to radiologists in detecting PEs. The integration of AI-based tools in PE detection shows strong potential when implemented alongside radiologists. This collaborative approach can enhance diagnostic accuracy and efficiency. The typical imaging modalities used in these studies focused on CT pulmonary angiography, known to be the gold standard in the detection of PE because of its high sensitivity, specificity, accuracy, and wide availability. Model designs and their use of different architectures, frameworks, and deep learning must be adapted for a very specialized diagnosis of PE on radiological imaging effectively. The use of segmentation models like U-Net, R2Unet, and Retina U-Net, as well as classification models (e.g. DenseNet201 and EfficientNet), suggests varying levels of specialization for detecting features of PE, in addition to requiring effective fine tuning for each model and dataset. This poses a problem of generalizability to new data inputted into the models, which is seen in the variability of the detection accuracy seen in ranging from roughly 68% to almost 100%. Some factors accounting for the variation seen are the quality of the dataset (e.g. high- or low-resolution CT, the diversity of medical imaging data (i.e. harder to identify complex PE presentations), sample size (many datasets were split into a training set and a testing set, which further decreased the sample size used to test model performance), whether the imaging has been annotated or not and the quality of model design (e.g. using multihead attention and dual-hop). In terms of sample size, more diverse studies with larger datasets tend to achieve more trustworthy findings and generally perform better in actual applications, such as Khan’s and Condrea’s studies. In addition, the inclusion of clinical or lab results is mentioned as a potential way to better train a model to identify or accurately diagnose PE from patient clinical data in addition to imaging [52-73].

Some limitations in these studies are seen where Huang et al., Ajmera et al. Gotta et al. and Huhtanen et al. applied datasets from only one academic institution, restraining the applicability to the general population. Similarly, Khan only used a single dataset however, the volume of the dataset was vast and diverse. Nevertheless, computational complexity due to the extent of the population was another limitation. The Topff et al. study mentions the findings of multiple types of bias, from patient population bias, the study was focused on oncology patients; selection bias, the data sets were not all-inclusive; labelling and annotation bias; among others. They emphasize the feedback loop bias, in the context of this study; it happens when radiologists may become reliant on the output of the AI at the risk of probably missed diagnoses when a case is flagged numerous times. The temporal bias is also stressed upon since demographics, clinical practices, and imaging are constantly changing and this would impact the efficacy of the AI at some point in time. Additionally, Huhtanen et al. and Lanza et al. mentioned limitations in locating the exact location of the emboli and detecting PE in uncommon complex cases. This may be improved by adding an extra stage of training to make the model more anatomically aware, and as Condrea et al. mentioned it may increase the performance of the model overall. Overconfidence in the AI models among healthcare specialists can be life-threatening without human supervision since misclassification of certain conditions or misdiagnosis happens occasionally even among very well-performing models. The potential use of AI as a tool for diagnosing PE in real-life clinical practice could be revolutionary by reducing the time taken to interpret images, leading to rapid diagnosis and quick reporting. Furthermore, AI decreases the radiologist’s workload, since models can process large volumes of data, enabling them to concentrate on elaborate cases and other priority tasks. Most of the studies, despite various diverse datasets, demonstrated potential for real-world application, just as in the case of Huang et al. as a triage tool. The case of the Gotta et al. study, which used a GBT model for the prognosis of PE also displayed a high accuracy in PE detection but due to the limited sample size, the generalizability of the study on actual application will be restrained. Continuous and robust testing of AI models across a wide range of clinical settings to ensure effective performance across myriad real-world scenarios. Comparing how the AI-based tool individually performs in comparison to individual radiologists to better appreciate the value added by the AI system itself. Quantitative evaluation of images in the present dataset and the proportion of optimal versus suboptimal CT pulmonary angiography (CTPA) scans as AI-based tools work better on optimal imaging conditions. Prospective studies in real-world conditions will help provide better insights into how AI-based tools can improve diagnostic accuracies and outcomes for patients. The integration of AI tools in clinical workflows to avoid missed or delayed diagnoses and better healthcare delivery and patient care. There is much promise for AI to be useful in automating medical imaging for better diagnostics and saving time, while several challenges still remain to make it reliable and trustworthy. Hence much like there are gold standards in treatment, guidelines for a trustworthy model for clinical use need to be developed. In the past, CAD algorithms designed for the detection of PE using CTPA scans had a relatively low sensitivity level. Researchers have developed a deep learning model with U-Net architecture showing good performance on an external dataset but underlined the need for further work to enhance the interpretability and trust of such models. Studies also highlight that engagement in the human decision-making process along with AI may even accelerate the triage of PE since diagnosis and therapy might get closer to each other. They also report that AI may identify specific positive scans to be reviewed in just a couple of seconds by flagging them as potentially abnormal [52-73].

## Conclusions

Pulmonary embolism is a challenging diagnosis and AI tools and models can contribute to improving the diagnostic efficacy and clinical outcomes in PE. These benefits may prove to be crucial in situations where prompt diagnosis and management are essential to prevent associated morbidity and mortality. Some of the AI tools have demonstrated similar or superior sensitivity and specificity for identifying PE as compared to sole interpretation by radiologists. However, it is imperative to design more sophisticated software and conduct clinical trials with larger sample sizes to integrate these tools into everyday clinical use and guidelines. Additional scientific evidence backing up the use of AI will facilitate its transition from a developing research tool to a routinely used, high-yield aid for physicians and enhance patient outcomes.
